# The Role of Surface Chemistry in the Efficacy of Protein and DNA Microarrays for Label-Free Detection: An Overview

**DOI:** 10.3390/polym13071026

**Published:** 2021-03-26

**Authors:** Elisa Chiodi, Allison M. Marn, Matthew T. Geib, M. Selim Ünlü

**Affiliations:** 1 Department of Electrical Engineering, Boston University, Boston, MA 02215, USA; ammarn@bu.edu (A.M.M.); geib@bu.edu (M.T.G.); selim@bu.edu (M.S.Ü.); 2 Department of Biomedical Engineering, Boston University, Boston, MA 02215, USA

**Keywords:** polymeric coating, microarrays, surface chemistry, label-free sensors, binding kinetics, single-particle imaging

## Abstract

The importance of microarrays in diagnostics and medicine has drastically increased in the last few years. Nevertheless, the efficiency of a microarray-based assay intrinsically depends on the density and functionality of the biorecognition elements immobilized onto each sensor spot. Recently, researchers have put effort into developing new functionalization strategies and technologies which provide efficient immobilization and stability of any sort of molecule. Here, we present an overview of the most widely used methods of surface functionalization of microarray substrates, as well as the most recent advances in the field, and compare their performance in terms of optimal immobilization of the bioreceptor molecules. We focus on label-free microarrays and, in particular, we aim to describe the impact of surface chemistry on two types of microarray-based sensors: microarrays for single particle imaging and for label-free measurements of binding kinetics. Both protein and DNA microarrays are taken into consideration, and the effect of different polymeric coatings on the molecules’ functionalities is critically analyzed.

## 1. Introduction

Microarrays are ordered collections of molecules deposited on a surface in small spots [1,2]. The first example of a microarray was introduced by Gergen et al. in 1979, and consisted of recombinant DNA plasmids that were deposited on filter paper, then hybridized with specific cDNA sequences [3]. Due to the rising popularity of the field of genomics, DNA microarrays were the first to be developed, addressing the need to keep track of many DNA sequences [4]. To this day, DNA microarrays are a well-established method to detect DNA mutations and are widely used in cancer research and diagnosis [5,6,7].

In the recent years, however, protein microarrays have also gained popularity as an irreplaceable tool for the fields of diagnostics and drug development, as they constitute an efficient method for multiplexed detection of biomarkers and antibodies [8,9,10,11,12]. Protein-protein interactions impact every aspect of the human life, from immune response, to enzymatic inhibition [13]. Proteins and antibodies are widely characterized with many different methods, but label-free techniques allow for dynamic measurement of binding affinity constants [14]. Label-free techniques provide direct assessment of the biomass that is accumulated on the surface, allowing for precise quantification up to the number of captured molecules [15,16], which would be impossible with a labeled technique such as fluorescence, where the need for a secondary fluorescent molecule prevents the possibility of obtaining a direct correlation of the fluorescence signal with the amount of accumulated biomass.

Antibody and protein microarrays have a giant research and diagnostic potential. One of the main research areas currently undergoing exceptional growth is detection and characterization of small molecule compounds, essential for the drug development process. In 2019, more than 70% of the American Food and Drug Administration (FDA) approved drugs were categorized as small molecules [17], defined as the ensemble of chemical compounds with a molar mass below 1 kDa [18]. Small molecules are usually characterized by measuring their binding affinity to distinct groups of many antibodies, mostly through label-free techniques [19,20]. Given their small size, label-free characterization is challenging but preferred, since labeling such small compounds can be tricky. Small molecules do not possess many binding sites for an eventual label, and the steric hindrance caused by the presence of the label might inhibit the binding to the biorecognition elements. Still, the main challenge when performing label-free measurements of small molecule kinetics is the maximization of the signal level. Most label-free techniques rely on measuring refractive index changes at the substrate-liquid interface [21,22,23,24], and a small-sized target will cause a very small change in refractive index when bound. Therefore, maximizing the binding signal is crucial. Multiplexed antibody microarrays are normally utilized to perform such a characterization, once again highlighting the importance of protein microarray stability for drug development applications. In order to obtain precise characterization data, the antibodies need to be stable, active and the binding sites must be accessible by the target molecules. Surface chemistry plays a fundamental role in obtaining the most efficient capture surface possible, since the immobilization strategy influences both the structure and the functionality of the immobilized probes.

Additionally, biomarkers, such as hormones, cholesterol, but also bigger and more complex structures such as extracellular vesicles, provide understanding of the endpoints of biological processes, and their characterization is crucial to improve diagnostic methods. Extracellular vesicles, for example, have been shown to carry important diagnostic information such as cancer markers [25]. Characterization of biomarkers and their carriers is also carried out by capture onto antibody-functionalized microarray substrates. In the case of label-free characterization of biological nanoparticles, two approaches are mostly utilized. One possibility is single particle capture and imaging with atomic force microscopy (AFM) [26], scanning or transmission electron microscope (SEM, TEM) [27], plasmonic resonance or interferometric imaging [28,29]. Another approach is to obtain a bulk measurement of extracellular vesicles (EVs) mass accumulation, which has been demonstrated both by interferometric and plasmonic resonance imaging (IRIS and SPRi) [30,31]. For the matter of our discussion, we will mainly address SPR (or SPRi) and IRIS, since these methods make use of different molecular immobilization strategies. Many other label free methods have demonstrated biomarker detection and recognition capabilities with good sensitivity, such as Surface-Enhanced Raman Scattering (SERS) [32], and Reflective Phantom Interface (RPI) [33,34]. In particular, RPI has been utilized to detect Flaviviral antibodies in human serum samples, demonstrating its potential as a point-of-care platform [35]. However, comparison and evaluation of different label-free detection methods exceeds the scope of this discussion, which will focus instead on reviewing the most commonly utilized surface functionalization methods. Indeed, both SERS and RPI, as well as many other label-free techniques, typically apply some of the surface treatment methods that are broadly discussed here. In the case of SERS, the most common approach is to functionalize the nanoparticle-coated surface with a chemistry that is suitable for gold/metallic substrates [36], whereas RPI employs a perfluorinated amorphous copolymer cartridge that is then coated in copoly(DMA-NAS-MAPS), also addressed below.

In general, the functionality of microarrays is intrinsically dependent on the surface morphology, which influences both the sensitivity of the measurements and the reactivity of the biorecognition molecules. For nanoparticle detection, a rough surface could disguise the particles, impacting the detection capability. For specific molecular assays, the surface probes need to maintain their native functionality, therefore the molecular structure must be preserved. Moreover, they need to be spaced and distributed enough that the target can easily reach them. Maintaining a specific orientation of the immobilized molecules is also helpful in order to maximize binding [37]. Surface chemistry development is a research field that aims at efficiently immobilizing bioreceptors onto a rigid substrate, without excessively altering the surface morphology. Many different approaches are utilized for this purpose, from coating the surface with epoxisilane-based polymeric thin films [38,39] or with matrix-structured polymers whose tridimensional properties contribute to preserving the structure and functionalities of the molecules [40,41,42], to the creation of nanostructures [43] which, among other things, enable specific control of the wettability of the surface [44,45].

**Figure 1:**
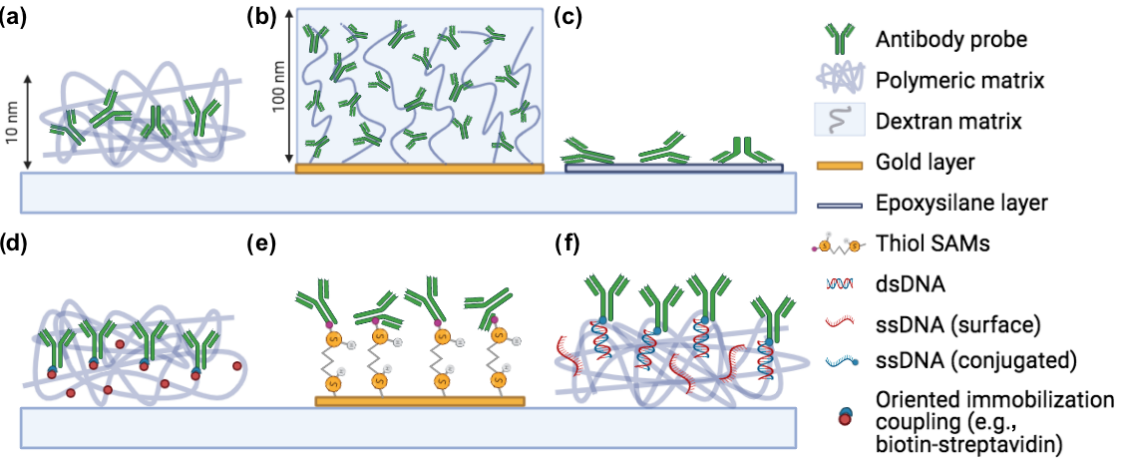
Common immobilization methods for microarray applications. (**a**) Copoly(DMA-NAS-MAPS), (**b**) Carboxymethyl dextran, (**c**) Epoxysilane, (**d**) Oriented immobilization (biotin-streptavidin, click chemistry), (**e**) Thiol-gold coupling, (**f**) DNA-directed immobilization. Created with Biorender.com, accessed on 1 January 2021.

In this review, we describe the impact of surface chemistry on the efficacy of label-free microarray-based assays and imaging sensors. We will consider both established technologies as well as newly developed strategies. The focus of this work will be on label-free sensors, both applied to single particle imaging as well as to binding kinetics affinity measurements. We will have a section dedicated to each method, comparing the effect of different functionalization techniques on the sensitivity of the sensors, as well as on the molecules’ functionality. The main immobilization methods described in this review are summarized in Figure 1.

## 2. Popular Functionalization Methods

### 2.1. Types of Surfaces and Biorecognition Elements

Traditionally, microarrays are printed on glass surfaces. However, other surface types can be adapted as microarray supports, and choosing different materials necessitates tailored surface functionalization methods. Silanes, for example, are most commonly used to functionalize glass surfaces, as further discussed in Section 2.3. Gold surfaces, on the other hand, which have become hugely popular since the advent of Surface Plasmon Resonance (SPR), are usually activated with thiols. Further details on these chemistry-surface pairings are given in the next sections. Other materials that can be coated and activated include cellulose [46], nitrocellulose [47,48] and plastic [49]. Nitrocellulose possesses a naturally high binding affinity for proteins, DNA, and RNA molecules, which bind in an irreversible manner to the film through a combination of hydrophobic interactions and silanization. The details of this mechanism, however, are still not fully understood [48], and coating with an additional polymeric layer enhances and controls its immobilization capabilities [47]. Plastic microarrays have the advantage of being both cost effective and malleable, allowing for the patterning of microfluidic systems on the same support. Moreover, there is no need for an additional coating, since the material itself is usually an active polymer containing carboxylic acids, which can be activated to form N-hydroxysuccinimide (NHS) esters that in turn react with amino groups on the molecules, providing immobilization.

As different surfaces require tailored activation protocols, molecules with diverse functionalities also require customized immobilization strategies. For example, antibodies and proteins contain a high abundance of amino groups, and are therefore commonly immobilized by reaction of those groups with NHS esters. On the other hand, DNA and peptides are synthetically produced and can be easily modified during the synthesis phase with specific groups, such as thiols, DBCO, azido groups or biotin. The working principle and the importance of each of these chemistries is discussed in the following sections. This flexibility allows for more freedom when it comes to immobilizing DNA or peptides, with respect to proteins. Modifying proteins with an additional group is still possible, though with potential effects on their reactivity.

In the next sections, we will discuss different immobilization methods and their implications. For each technique, we will specify the surface material, bioreceptor type and efficacy. Table 1 summarizes the limits and advantages of the immobilzation techniques discussed in this work.

### 2.2. Physisorption: Spontaneous Adsorption on the Surface

Physical adsorption is the simplest and most straightforward immobilization method for any application. It exploits the weak electrostatic and Van der Waals interactions that form between the studied biomaterial and the first atomic layers of the support in order to weakly anchor the biomolecules to the surface. This method does not require any modification of either the molecule or the surface [38], and it can sometimes be used for DNA, taking advantage of the negative overall charge which characterizes these molecules. However, when immobilized with this method, the DNA probes lay completely flat on the surface, limiting possible conformation changes and distorting the molecular structure. Moreover, detergents—often necessary in buffers for DNA experiments—contribute to desorbing the molecules from the surface [38]. Enzymes, on the other hand, are more likely to be immobilized by physical absorption [60,61]. This technique remains not ideal, since—due to the weak nature of such interactions—changes in ionic strength, temperature or pH might easily desorb the molecules from the surface. Therefore, new solutions such as immobilization on polymers and sol-gels have been explored [61]. Atomic Force Microscopy measurements have been used to compare covalent immobilization of enzymes and physical adsorption [62]. Immobilization of unmodified DNA in agarose gels has been also been demonstrated by using UV light [53].

### 2.3. Polymeric Coatings

Polymeric coatings are the most commonly used methods to immobilize molecules onto a solid substrate [38]. The quality of a polymeric coating can be evaluated by considering the amount of biomass that is stably anchored to the surface, as well as the functionality of the molecules that have been immobilized. In general, functionalization of the surface with an active polymer is performed in multiple steps. First, the bare surface needs to be activated in order to expose reactive groups. Then, the polymer is allowed to react with the surface for a variable amount of time, forming a uniform and stable coating. Finally, the biomolecules are deposited on the surface and chemically react with the functional groups of the polymer, and immobilization is achieved.

Epoxysilane-based polymers are extremely reactive polymeric coatings widely employed in the field of biosensors. Their ease of use, rapidity and low cost constitute the main reasons for their broad popularity. However, traditional epoxysilane also presents a number of disadvantages, potentially constituting a limiting factor for certain applications.

The working principle of epoxysilane polymers is based on the creation of a self assembled monolayer (SAM) of oriented epoxide groups, a task performed by two functional ends. One end contains silane groups which covalently bind to silica substrates, while the other side contains an epoxide group which is reactive to primary amines or other groups on the surface of biomolecules [63]. One of the most commonly used epoxysilane based polymers is (3-Glycidyloxypropyl)trimethoxysilane (GLYMO) [64]. The substrate of choice when utilizing silane-based polymers is usually glass, due to its reactivity with silicon oxide molecules. However, silicon slides with a thermally grown silicon oxide layer on top have also been used for this application [23], as thin film interference has been utilized for label-free applications, as well as to enhance fluorescence signal for microarray readers [65,66]. Silane-modified glass shows high binding efficiency [67]. However, this type of immobilization can sometimes lead to coffee ring effects [45] and other non-homogeneity issues, sometimes requiring the addition of glycerol or other additives in order to normalize spot morphology [67]. Moreover, the silane coating is essentially bi-dimensional, forming a very thin layer that does not tend to preserve the molecular structure of the immobilized biomolecules. As a matter of fact, by stretching in a single layer on the surface, the molecular structure is deformed, affecting the protein stability and functionality [68].

**Figure 2:**
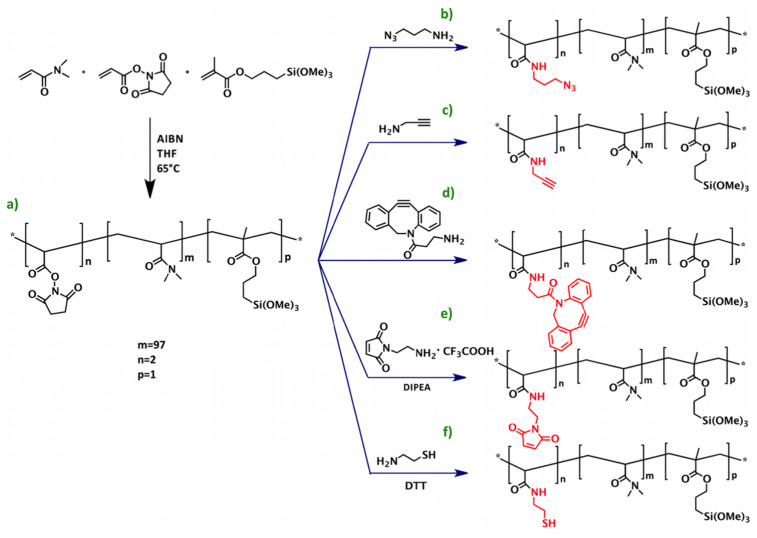
Scheme of the synthesis of copoly(DMA-NAS-MAPS) (**a**) and its derivatives obtained through post-polymerization modification (PPM), copoly azide (**b**), copoly alkyne (**c**), copoly DBCO (**d**), copoly maleimide (**e**), and copoly thiol (**f**). Here AIBN is a short for Azobisisobutyronitrile, THF for Tetrahydrofuran, DIPEA for N,N-Diisopropylethylamine, and DTT for Dithiothreitol. The asterisk in inset (**a**) indicates where the functional groups are being linked. Reprinted with permission from [69], copyright (2016) American Chemical Society.

As an alternative to traditional 2D-epoxysilane coatings, NHS-based reactive polymers that form a tridimensional matrix when hydrated are widely used in the microarray research field. An example is the copoly(DMA-NAS-MAPS) [40]—commercially known as MCP-2 - and the related family of polymers obtained from the latter by post polymerization modification (PPM) [54,55,69]. These polymers have the ability to easily form thin film layers on most materials by utilizing a combination of physical and chemical adsorption, which is particularly efficient on silica substrates. Trimethoxysilane moieties confer to the polymers the ability to form stable bonds with the oxide groups on the surface, while anchoring of the bioreceptors is possible through binding of the active succinimidyl esters groups with the free amine groups on the molecules, achieving covalent immobilization through an amide bond. PPM allows to introduce functional groups such as azide groups [54,55,69], as shown in Figure 2.

**Figure 3:**
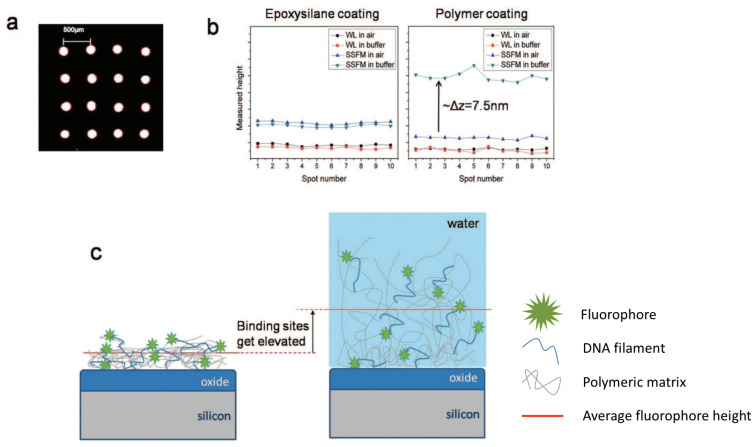
(**a**) Fluorescence scan of a typical array of ssDNA spots on copoly(DMA-NAS-MAPS). The spots are ≈150 μm in size with 500 μm pitch. (**b**) Detected heights with WL and SSFM techniques for 10 different spots on the same sample before andafter hydration. The epoxysilanized sample shows no change in White Light reflection spectroscopy (WL) and Self-Interference Fluorescence Microscopy (SSFM) levels before and after hydration. For the polymer coated sample, the WL level is maintained upon hydration, whereas the SSFM level increases by ≈7.5 nm. (**c**) Illustration of the justification for the height change in probe heights immobilized on the polymer. The polymer swells upon hydration, resulting in elevation of the binding sites. Figure adapted from with permission from [68]. Copyright (2009) American Chemical Society.

It has been extensively demonstrated that such polymers provide uniform immobilization of both proteins and amine-modified oligonucleotides [40]. Moreover, when immersed in water or saline solutions, these coatings form a tri-dimensional matrix structure on the surface, elevating the probes and preserving the molecular structure. This capability has been demonstrated through a combination of fluorescence and label-free measurements, where the distance of a single fluorophore from the surface could be measured [68]. In this work, immobilization of DNA molecules on MCP-2 and silane-modified Si/SiO_2_ slides is performed, and the efficiency of the two techniques in terms of both spot homogeneity and molecular activity is compared. The results provided demonstrate that the tri-dimensional structure provided by MCP-2 allows the DNA molecules to maintain their rigidity and better hybridize with the complementary sequence. The fluorophores are elevated at around 10 nm from the surface when the co-polymer is hydrated, and at 2 nm when it is dry, while for silane no difference in height of the molecules is measured, proving that molecules immobilized on silane are flat on the surface and lose their structure (Figure 3).

While similar experiments have not yet been performed to compare the efficiency of the two chemistries for protein microarrays, other works show the improved performance of 3D-matrix forming chemistries in the immobilization of proteins with respect to silane bidimensional coatings [39].

Dendrimer-based polymers have also been showed to efficiently immobilize DNA [50]. A step by step method is proposed where silanization is performed first, followed by chemical activation and attachment of linear crosslinkers, and finally by covalent binding of a thin polymeric layer of dendrimers, which are then amino-coupled to DNA molecules. The main advantage of this method is the resistance to harsh regeneration protocols, allowing for reusable DNA microarrays.

### 2.4. Nanostructure-Based Methods

Glass and silicon slides are a common choice as substrates for microarray deposition. Their low autofluorescence makes them suitable for fluorescence measurements. On the other hand, for what concerns label-free measurements, a thin layer of a different material is usually manufactured on the slides (thermally grown silicon oxide on silicon, or evaporated gold on glass), which—depending on the technique used—provides the resonance or the enhancement needed to detect the binding signal.

In order to efficiently immobilize bioreceptors onto these substrates, an alternative to polymeric coating is to mold the existing thin layer of material into nanostructures. As mentioned above, tridimensional active structures allow for maintaining the reactivity of the molecules, while also possessing higher loading capacity, thanks to the more uniform distribution of the molecules throughout the substrate [45].

**Figure 4:**
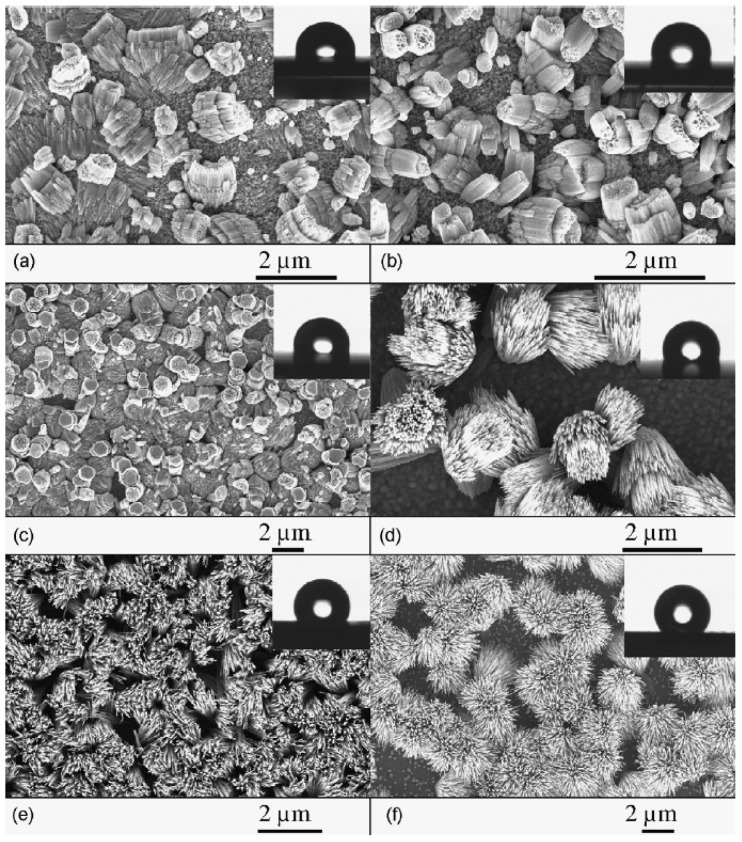
SEM images of ZnO nanostructures as a function of growth temperature (Tgr): (**a**) corals (Tgr = 200–240 ${}^{\circ }$C), (**b**) cabbages (Tgr = 240–280 ${}^{\circ }$C), (**c**) porous hexagons (Tgr = 280–320 ${}^{\circ }$C), (**d**) bundles (Tgr = 320–365 ${}^{\circ }$C), (**e**) sheaves (Tgr = 365–440 ${}^{\circ }$C) and (**f**) open sheaves (Tgr = 440–550 ${}^{\circ }$C). The insets show contact angle images from droplet experiments for the respective as-grown samples, which demonstrate size-dependent, tunable hydrophobicity. Figure reproduced with permission from [44]. Copyright (2012) Elsevier.

Nanostructures have been utilized for improving immobilization efficiency, from nanopillars and nanotubes [70,71] to porous surfaces [72]. One of the main advantages of nanostructures for microarray applications is the ability to control the wettability of the surface, creating super-hydrophilic and super-hydrophobic regions as needed [44,73], as shown in Figure 4 for differently sized ZnO nanostructures.

Nanostructured polymers on glass slides have succesfully been utilized to immobilize antibodies and proteins in a microarray modality, achieving high loading capacity and better spot morphology with respect to epoxysilane slides [45]. In the label-free field, gold nanostructures are often employed for surface plasmon resonance (SPR) and localized SPR measurements [74,75]. Such nanostructures, however, are not reactive to the biomolecules and still need to be functionalized following the surface chemistry approaches used for gold (thiol SAMs, etc.). The experiments are discussed in more detail in Section 3.3. Nanoholes substrates have been successfully employed for plasmonic detection of both molecular analytes [76] and of single nanoparticles, such as exosomes [28] in transmission-based SPR sensors. Here, both the structure and the specificity of exosomes is evaluated, by combining single particle imaging with capture by breast cancer markers. Clear discrimination between healthy and cancer patients is demonstrated.

A worth-mentioning, non-microarray, label-free application of nanostructures is for electrochemical sensing. For example, lypopolisaccarides dose–response curves were obtained with oxide-based nanostructured electrodes [77].

### 2.5. Other Methods

One of the first immobilization strategies developed for DNA microarrays was by photopolymerization of μm-sized polyacrilamide gel pads immobilized on a hydrophobic glass surface [51]. The approach was also successfully applied to proteins, once again demonstrating high load capacity and higher binding signal for tridimensional structures with respect to bidimensional solutions [52]. However, the long and cumbersome preparation of the chips, together with the particular consistency and refractive index of gel, make the application of this method to label-free measurements impracticable. The surface is hardly recoverable, due to the difficulty in changing buffer from inside the gel, so that dose–response curves are technically unobtainable. The method is—in fact—mostly applied to photoluminescence and fluorescence endpoint measurements. Even in that case, the viscosity of hydrogels reduces the dynamics of the reaction, limiting the sensitivity of the measurements [78].

Another widely employed immobilization method is based on the biotin-streptavidin interaction [38], one of the strongest binding interactions in nature, second only to the covalent bond [79,80]. This immobilization technique requires modification of both the surface—usually, streptavidin coated—and the molecule—biotin-modified. While for proteins and antibodies amine-binding polymers are normally more ideal, since they naturally carry amine groups, for DNA, modification with an amine group would be required, and therefore, biotinylation of DNA is often used. While the streptavidin-biotin interaction is very hard to break, depending on the chosen substrate, streptavidin desorption from the surface might occur, resulting in probe loss. Protein A and protein G are also widely utilized, given their high affinity to the Fc portion of mammalian antibodies. While streptavidin-biotin allows for oriented immobilization, though, protein A and protein G provide random immobilization, but have the advantage of not requiring modification of the probe molecule. Streptavidin, protein A and protein G—however—still need to be immobilized on the surface, requiring additional surface activation [58].

## 3. Microarray Types and Specific Immobilization Strategies

### 3.1. Microarrays for Label-Free Single Particle Imaging

Single-particle label-free imaging is widely employed for visualization of viruses [29], exosomes [28,81], and bacteria [82] as well as for other synthetic and biological nanoparticles [83,84].

One of the main challenges when imaging single biological micro- and nano-particles is the capture efficiency of the active surface [85]. For the purpose of this discussion, we will focus on the capture and imaging of whole biological and synthetic particles onto multiplexed protein microarrays. Naturally, rigidity and density of the bioreceptors influence the capture efficiency of the spots. As a matter of fact, the probe array must be dense in order to provide enough binding sites to stably bind the particles, while also not being too packed, to avoid crowding and steric hindrance effects. A possible solution to this issue is to choose very flexible receptors, which better adapt to the structure of the nanoparticles. As a consequence, the number of possible spatial configurations in which the particles can be captured with high efficiency increases. In general, smaller capture agents, such as peptides or aptamers, are a good solution, due to their small, non-rigid structure. However, flexible probes are not always available for every application. Most of the time the biorecognition elements are antibodies possessing a large, rigid structure. In that case, utilizing DNA filaments to improve probe flexibility can be a good solution, as further discussed in the following section.

#### 3.1.1. DNA-Directed Microarrays

DNA-directed immobilization (DDI) of antibodies and proteins is a highly efficient technique for generating patterns of oriented molecules on active surfaces [56]. Since its first introduction in 1994 [86], it has been utilized and adapted to various applications, from multiplexed antibody-based immunoassays to cell arrays [56,57,87].

**Figure 5:**
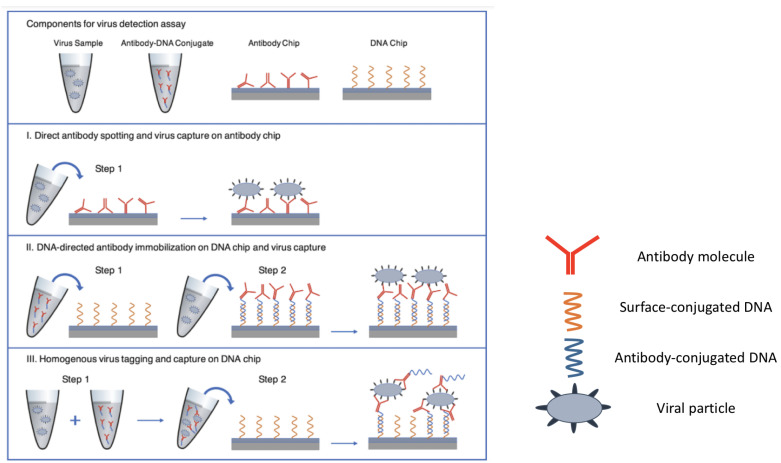
Three different approaches for virus capture on the SP-IRIS chip surface. (I) Direct antibody spotting (II) DNA-directed antibody immobilization (III) Homogenous virus tagging in solution with antibody-DNA conjugates. Figure adapted with permission from [88]. Copyright (2021) American Chemical Society.

In this approach, single stranded DNA (ssDNA) sequences are immobilized on the chosen substrate through a reactive polymer (usually, amine-based). Concurrently, monoclonal antibodies against membrane or shell proteins of a specific biological nanoparticle are modified with the complementary ssDNA sequence. Incubation of the substrate with the DNA-directed antibody solution is then performed, in order to immobilize the DNA-directed probes on the surface. Finally, the substrate is incubated with the particles sample, to achieve capture, and is then analyzed with the method of choice. Sometimes the incubation and capture are performed simultaneously and detected in real time [88], as represented in Figure 5 for homogeneous virus detection on DNA microarrays.

One of the advantages of the DDI approach is the long-term stability of the arrays. DNA microarrays are highly stable, both physically and chemically. Thus, the substrates can be mass produced and utilized over a long time span, on the order of months. This is not possible with standard protein microarrays, which degrade in a much shorter time [89]. DDI microarrays therefore match all the advantages of protein microarrays with the stability of DNA microarrays.

The DDI method has been successfully applied to single particle capture. Particularly, improved capture efficiency has been shown, due to the added flexibility to the probes [81,90]. The binding of DNA-directed antibodies to the nanoparticles and of the nanoparticles to the substrate can also take place by self-assembly, where all the incubation steps are performed simultaneously [91]. The antibodies can also be incubated with the particles prior to capture, changing the order of the binding reaction with respect to the standard methods [88]. Both these approaches demonstrate high efficiency with shorter incubation times, however, they provide less control of non-specific binding.

Another advantage of the DDI approach is the possibility to recover the captured particles by cutting DNA strands with DNAse solutions [81]. In some cases, the biotin-streptavidin interaction is also employed as a means to ensure stable binding [92]. Cell-sized DDI features can also be created for single mammalian cell encoding and capture [93].

#### 3.1.2. Peptide Microarrays

Peptides are the building bricks of proteins. Thanks to their small size and versatility, they have now become standard discovery tools, and have great potential for development in diagnostics. To date, they have been used in many applications, from epitope mapping to antibody profiling [94]. Recently, membrane binding peptides that are specific to small extracellular vesicles have been introduced [95], which target the high curvature and the membrane defects typical of small EVs. Since the capture is highly specific for small EVs, but non-specific for tetraspanins or other surface markers, these peptides are ideal as positive control in single vesicle characterization experiments. Peptides specific against a number of viruses and virus antibodies have also been introduced, for example zika-specific peptides show 95% accuracy in serological assays [96].

Commonly, peptide arrays are either synthesized directly on the solid support utilized for the measurements by light-directed synthesis [97] or they can be spotted and immobilized on a polymer-coated surface via amide coupling or epoxide ring opening [94]. However, an efficient method to immobilize peptides in an oriented fashion is through click chemistry [54,55,98]. The term ‘click’ is used to indicate chemical reactions that show high conversion yield in short reaction times [99]. Copper(I)-catalyzed [3 + 2] azide–alkyne cycloaddition (CuAAC) has been shown to achieve oriented immobilization of peptides in a microarray format [54]. The standard copoly(DMA-NAS-MAPS) is modified by post-polymerization modification (PPM) to introduce azido groups that enable the CuAAC reaction. Similarly to the biotin-streptavidin immobilization approach, the CuAAC method allows for oriented immobilization, by specifically targeting dibenzocyclooctine (DBCO) groups on the molecules, which can be introduced in peptides during chemical synthesis. Click chemistry can be applied to any molecule that contains a DBCO group; therefore, it can be easily exploited for immobilization of DBCO-modified DNA and proteins. Oriented immobilization allows to precisely know which part of the molecule is exposed to the target solution, and - conversely - which part is unavailable for binding, being involved in surface attachment. It has been demonstrated that oriented immobilization can dramatically improve binding efficiency, yielding a signal more than ten times higher with respect to random immobilization for peptides binding to an SPR sensor [37].

### 3.2. Protein Microarrays for Binding Kinetics Assays

Binding kinetics assays are widely employed as a means of characterizing the affinity of multiple biomolecules simultaneously. With respect to ELISA or lateral-flow assays, kinetic measurements provide the user with information regarding the dynamics of the binding reaction, which can sometimes be more comprehensive with respect to equilibrium, end-point data. Moreover, the absence of a label facilitates the determination of the inner properties of the compound, not influenced by the presence of a secondary agent. For these reasons, binding kinetics assays are very popular in the field of drug development and antibody research, where multiplexed substrates are scanned against the target of interest.

As for other applications, the ideal substrates for binding kinetics measurements will have a uniform surface morphology, a high loading capacity as well as preserving the molecular activity of the molecules. Moreover, cross reactivity needs to be minimized, especially for multiplexed experiments where many biorecognition elements are immobilized on the same substrate, and specific binding maximized, to characterize small sized analytes as well as low concentrated samples.

The most popular and widely employed technique in biomedical research to perform binding kinetics measurements is Surface Plasmon Resonance (SPR). Standard SPR instruments call for even more restrictive surface conformation requirements, including for example the need for a chemistry that minimizes non-specific binding. Compared to its imaging counterpart, SPR imaging (SPRi) and other label-free imaging techniques, SPR is a non-selective detection method [100], thus non-specific and specific binding are impossible to discern. Therefore, it is crucial to utilize an immobilization technique which completely eliminates or at least reduces non-specific binding to a negligible amount. On the other hand, techniques based on microarray imaging are advantageous in this sense, since they allow for an easy monitoring of both the background around each spot and well-positioned, purposely inactive (negative) regions or spots. Carboxymethyl dextran (CM5) polymer layers are usually chosen for SPR experiments, thanks to their ability to form a tridimensional matrix to trap the molecules, again by amide bonds. Hydrophobic linkers are sometimes utilized in order to facilitate the access of the immobilized molecule to the fluid stream, but caution must be used when positioning the linkers to avoid the remaining active sites of the polymer, in order to reduce non-specific interactions [21]. Standard SPR experiments do not belong to the category of microarray-based measurements, since the whole channel is coated with the bioreceptor under study. We will therefore shift our focus to SPRi and other SPR techniques that exploit multiplexed immobilization.

Even though it is not as critical as for standard SPR, minimizing non-specific binding is fundamental for any kinetic technique. Quantifying non-specific binding can be challenging, and therefore, surface-treating procedures have been developed to minimize it to the point where it can be considered negligible. In general, three types of non-specific binding shall be considered: during the spotting phase, non specific binding of the bioreceptors to the surface chemistry; during the kinetic experiment, binding of the analyte molecules in regions outside the functionalized areas, and also non-specifically to the spotted areas.

One solution to avoid non-specific attachment of the spotted molecules to the surface chemistry is the use of anti-fouling polymers [101]. Anti-fouling coatings inhibit the spontaneous accumulation of material on the surface thanks to their neutral charge and hydrophilic properties. Sometimes PEG (Poly(ethylene)-glycol) or OEG (Oligo(ethylene)-glycol) chains are added to the coating in order to further reduce superfluous adsorption of biomaterial [102,103], a solution that might present some drawbacks, including spontaneous oxidation in physiological environments [103]. In general, surface charge can affect the loading capacity of a protein microarray, and utilizing positively or negatively charged chemistries might increase the local mass density by electrostatic retention of biomaterial. However, such interactions are weak and could cause probe loss when flow is involved. Most importantly, the activity of the molecules could be affected due to distortion of the molecular structure. In a recent work, we studied multiple surface chemistries simultaneously by creating localized, differently functionalized regions on the same support, and we showed how charged surfaces do not present, in fact, a great advantage with respect to neutral ones for protein-protein interaction [104].

In the case of immobilized single-stranded DNA, where the linear structure of the strand is crucial in order to favour hybridization, the issue of probe-surface interactions that could cause distortion is very delicate. These interactions have been proven to reduce duplex formation, and thus the measured hybridization affinity, even when using anti-fouling polymers [102,105,106]. It has been demonstrated that the affinity of DNA-DNA interactions in solution is much higher with respect to the situation where one strand is anchored to a surface [102,105], due to a combination of molecular crowding, surface-probe interaction, and repulsive force of the high-density probe spot [106]. Tuning the surface properties in order to limit such interactions could therefore be advantageous when running DNA hybridization experiments.

For what concerns the minimization of analyte molecules binding to the regions outside the spotted areas during the real-time measurements, a procedure can be performed prior to the experiments which inactivates the polymer reactive groups that are not involved in immobilization of the bioreceptors. Ethanolamine solutions are often utilized for this purpose, at various concentrations and pH levels [107,108,109]. Highly concentrated solutions of proteins such as Bovine Serum Albumin (BSA) can also be utilized. By adsorbing to the non-functionalized regions of the sensor surface, BSA prevents further accumulation during the binding experiments.

Finally, the analyte molecules could non-specifically attach to the target spots. This is the most complex circumstance to troubleshoot, since the non-specific interaction is in principle indistinguishable from the specific one. As mentioned above, one method could consist of monitoring ‘negative’ spots, that is, defined regions of the sensor where molecules that are not reactive to the studied analyte have been immobilized. Assuming that the electrostatic, weak interactions that cause non-specific binding are the same for both spots, it should be possible to determine if the analyte molecules are prone to bind in a widespread, unspecific manner.

For what concerns molecule dispensing, as an alternative to traditional robotic spotting, microarray preparation for SPRi sometimes features patterned microfluidics [100], obtained by building glass/plastic hybrid chips where many different bioreceptors are flowed simultaneously to achieve immobilization in microchannels or microwells. PDMS or another polymer is molded on top of glass slides to realize patterning. This method allows for very precise fluid dispensing and small chamber volumes (around 700 pL), but the preparation complexity strongly limits its widespread applicability. A more commonly utilized approach features microarray printing with robotic spotters. The methods for attaching capture molecules to SPRi chips are not very different from SPR, and range from modifying the molecules with a thiol group achieving direct binding to the gold surface [59], by utilizing self assembled monolayers (SAM) of alkanethiols, which exploit the same method to form stable bonds with a gold surface. The final layer terminates with free active groups (amine, carboxyl) which then bind the biomolecules. Moreover, NHS-treated surfaces are also very popular for SPRi sensors [110], due to their high reactivity to amine groups. Biotin-streptavidin immobilization is also fairly common, with the drawback of requiring SA/b modification of the target molecule [111].

### 3.3. Surface Chemistry for Small Molecule Kinetics

The use of amine-based polymeric coatings combined with interferometric imaging has allowed to achieve antibody characterization against common proteins [23,104], human dengue specific proteins [112], extracellular vesicles [31] and small molecules [20]. Small molecules are defined as the chemical compounds with a molecular weight below 1 kDa [18]. This category of molecules is one of the most challenging to characterize in a label-free manner, due to the small signal generated on the sensors [113], which requires minimization of noise in order to achieve a high signal-to-noise ratio. Our group has recently demonstrated characterization of fumonisin B1 (721.83 Da), common corn mycotoxin, across a 20-multiplexed antibody microarray surface [20]. Binding and debinding curves were acquired and affinity constants were measured for eighteen out of twenty antibodies. Signal processing methods such as different types of signal averaging were applied to reduce the noise and achieve the desired sensitivity, comparable to SPR [19] and improved with respect to SPR imaging [114]. In this case, MCP-2 polymer [40] was used as the functionalization method, and all antibodies were successfully immobilized homogeneously and achieving good spot morphology. Each bioreceptor was spotted at a different concentration due to different purification yields, yet the molecular activity was preserved for all the antibodies. Two bioreceptors resulted inactive: in one case, that is most probably related to insufficient purification yield, triggering insufficient immobilization, while for the other—successfully immobilized—degradation of the sample is the most plausible explanation. Nevertheless, a highly multiplexable sensor with small molecule sensitivity was demonstrated, with further potential for improvement.

**Figure 6:**
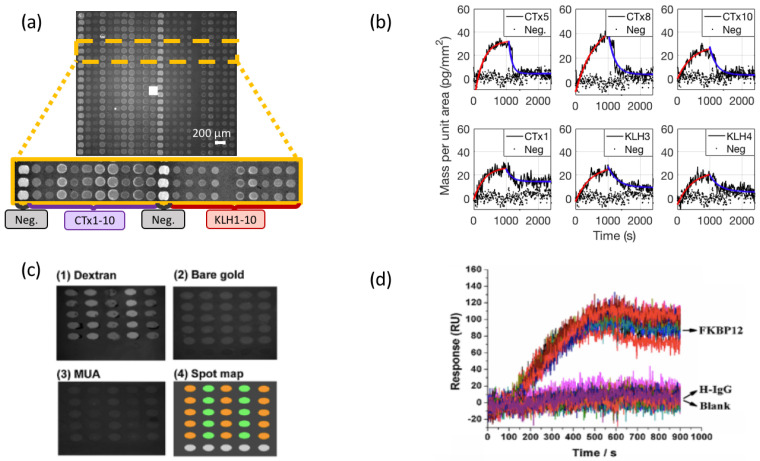
Small molecule characterization on the IRIS (**a**,**b**) and on the SPRi sensor (**c**,**d**). (**a**) Image obtained on the IRIS instrument for a multiplexed microarray spotted with 20 antibodies against fumonisin B1. The labels indicate the different antibodies immobilized on the surface (**b**) Set of six binding curves for fumonisin to the multiplexed surface acquired on the IRIS. The black lines (dotted and solid) represent the acquired data, while the red and blue line indicate the 1:1 association and dissociation Langmuir fit. (**c**) Image obtained on SPRi of an antibody microarray against FK506 spotted on different surfaces. The (1–3) insets shows SPRi images of the spots on a different surface chemistry, while inset (4) shows the spotting scheme. (**d**) Binding curves of FK506 acquired on SPRi. The arrows indicate which spots the data are obtained from. Parts (**a**,**b**) were adapted with permission from [20] (Copyright 2020 American Chemical Society). Parts (**c**,**d**) were adapted with permission from [115] (Copyright 2015 Elsevier).

A comparison of small molecule binding curves on the IRIS platform for fumonisin B1 (MW = 721.8 Da) and on an SPRi platform for FK506 (MW = 808.4 Da) [115] is reported in Figure 6, along with images of the microarray spots obtained on the two instruments.

Mycotoxin detection has been achieved on Localized SPR (L-SPR) platforms also, by immobilizing specific antibodies [74] and aptamers [75] onto 100 nm-sized islands of gold evaporated onto standard glass slides. Immobilization, in the case of antibodies, is performed by coating the nanostructures with multiple layers of polyelectrolytes using a layer-by-layer (LBL) approach. The utilized polyelectrolites are PAH (poly(allylamine hydrochloride)) and PSS (poly(4-styrene sulfonate)). After applying multiple PEs layers, Protein A is anchored to the surface through amide linkages [116,117] and finally a monoclonal antibody against the specific mycotoxin is immobilized. This immobilization technique is less then ideal: it requires multiple steps, which makes it complex and time consuming, and the stability of the bound antibodies can only be verified through a number of negative tests, further contributing to the complexity of the method [118]. On the other hand, aptamers were immobilized directly on gold by thiol coupling, guaranteeing stable binding, then activated through 5-min cycles of PCR. This method is much quicker and stable, and specificity of aptamers is extremely high [118]. In both cases, AFM measurements of the surface conformation are reported, showing the desired periodicity and uniformity of nanostructures. However, the nanostructures-based methods do not achieve the same sensitivity as continuous gold film SPR. Surface conformation could be one of the reason why high sensitivity is not achieved in this case. For example, the high density of nanostructures on the surface might cause steric hindrance, inhibiting binding. The same group succeeded in improving the sensitivity of the system by utilizing a similar approach where aluminum oxide gold-capped structures [43] are employed instead, showing an improvement in sensitivity when detecting thrombin down to pM concentration. Determination of affinity constants is demonstrated, however no real time binding curves are shown. Endpoint measurements are utilized to determine the equilibrium constant, since the system has yet to be integrated with a microfluidic setup.

A similar layer-by-layer approach is also possible by using click chemistry [119]. Such method allows for precise control of the thickness of the polymeric coating by covalent coupling of multiple layers of click polymers. The advantage of covalent coupling with respect to electrostatic bonding is its stability, which makes it insusceptible to changes in solution conditions such as pH or salt concentration.

## 4. Novel Technologies and Applications

Surface chemistry technology is in continuous development, due to the always growing need for robust and functional immobilization methods. One really promising technology has very recently been introduced which makes use of copper-free click chemistry reactions to immobilize fluorescent peptides on brush-like microstructures of a poly(ethylene) glycol (PEG) variant [120]. Spatial light modulation combined with photoinduced atom transfer radical polymerization (Photo-ATRP) allow to build hierarchical hyperbranched structures of two different polymers and finally immobilize fluorescent FITC-RGD peptides. This technique helps further reducing non specific binding by providing a surface with exceptional anti-fouling properties, but also—given the possibility of modulating the photo-induced growth of the polymer by changing the light pattern—it offers the possibility of creating customized 3D microstructures. Fibronectin, BSA and streptavidin have also been immobilized with the same method.

Zeolitic imidazolate frameworks (ZIF) are also worth mentioning. ZIFs are a subset of metal organic frameworks (MOFs), hybrid porous materials built as crystalline periodic networks of inorganic metal nodes joined by organic linkers [72,121]. One of the most attractive features of these material is the tunable size of the pores. MOFs have been used for adsorption and sensing, as well as catalysis, and ZIFs surfaces in particular are characterized by great stability in physiological conditions, and high loading capacity [72]. ZIF-8, for example, uses zinc as a metal node and 2-methylimidazole (HmIm) as a linker, and the encapsulation efficiency of bovine serum albumin (BSA) and insulin (Ins) into ZIF-8 was demonstrated to be above 75%. ZIFs can be synthesized via biomimetic mineralization, which uses a biomolecule as a directing agent. This way, the crystal is synthesized already encapsulating the molecule, eliminating the need for multiple steps [72]. ZIFs have not yet been applied to microarrays or to label free detection, but the technology is young and it might further develop on that front.

**Figure 7:**
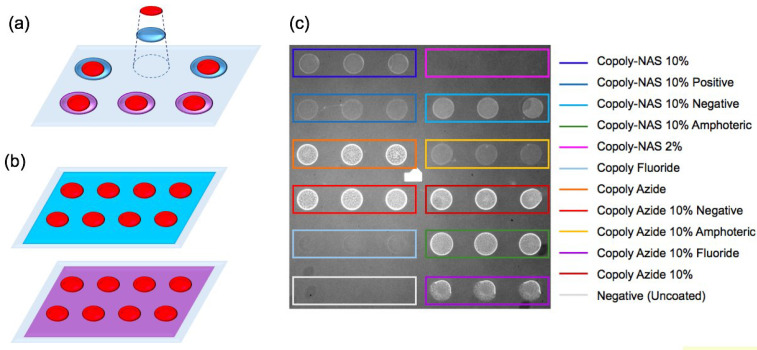
Localized surface chemistry technique. (**a**) On the left, a simplified scheme of the method compared to (**b**) the flat coating technique. The purple and blue surfaces represent different surface chemistries. The red circles represent spots of immobilized molecules. (**c**) On the right, an image of the chip acquired on the IRIS. On the chip, a 6 × 6 matrix of α-Lactalbumin spots is visible. Each group of three spots corresponds to a different polymer, as reported in the legend on the right. Figure reproduced with permission from [104] (Copyright 2019 Springer-Verlag GmbH Germany, part of Springer Nature).

Innovative approaches have also recently emerged in order to quickly characterize different types of surface chemistry. For example, our group has recently introduced a novel methodology to compare the performances of multiple surface chemistries simultaneously in order to establish the best one for each application. The technique is based on localized deposition of different polymers on the same substrate, which are then all functionalized with the same molecule, as shown in Figure 7, and finally scanned against a common target [104]. This allows to determine the probe density and activity of each surface, in a single experiment, consistently reducing the amount of time and materials that are spent choosing the right surface chemistry for each application.

## 5. Conclusions and Perspectives

Surface chemistry is one of the main factors that contribute to the success of label-free, microarray-modality experiments. Having an organized surface that is uniformly loaded with biorecognition elements, and that can resist changes in solution conditions such as pH and salt concentration, is fundamental in order to achieve good results, both for binding kinetics experiments and for single nanoparticle capture, detection and imaging. Table 1 summarizes the main molecular immobilization technologies that have been discussed in this review, along with their capabilities. We have already mentioned that surface chemistries that produce a tridimentional structure are generally preferred, since they have higher loading capacity and they preserve the molecular structure of the molecules, maintaining unaltered molecular activity. Furthermore, orientation plays a crucial role in maximizing binding reactions. Anti-fouling properties are needed in order to minimize non-specific interaction, and a smooth uniform structure is necessary when dealing with nanoparticle imaging. Such features, for the techniques discussed here, are summarized in Table 2. Organic polymeric coatings, such as copoly(DMA-NAS-MAPS), have demonstrated to be an optimal solution, satisfying most of these conditions. Their versatility and the possibility of adding functional group through post-polymerization modification is appealing for many applications. Moreover, it has been recently shown that molecules can be manually deposited and immobilized on such polymers without the use of a robotic spotter, which is convenient for laboratories that do not have such capabilities [122]. Other technologies such as nanostructures enable specific surface properties, like controlled wettability, while for some applications simpler methods are sufficient, such as direct coupling to gold for SPR or physical adsorption. Some of these methods can also be coupled in order to achieve the best configuration possible, such as immobilization of DNA-directed probes on organic polymers. The field is in continuous evolution, and new technologies are developed everyday. We believe that further development will allow to create surfaces that are even more efficient in order to further expand the use of label-free microarrays in medicine and diagnostics.

## Figures and Tables

**Table 1:** Main characteristics, capabilities and limits of the immobilization methods for label-free bioassays discussedhere.

Immobilization Strategy	Amide Coupling	Oriented	3D-Structure	Kinetics	Single Step	Probe Modification
Physical adsorption [38]	no	no	no	no	yes	no
Epoxysilane [38,39]	yes	no	no	yes	yes	no
Copoly(DMA-NAS-MAPS) [40]	yes	no	yes	yes	yes	no ${}^{\circ \circ }$
Dendrimers [50]	yes	no	yes	yes	no	no ${}^{\circ \circ }$
Hydrogels [51,52,53]	no	no	yes	no	no	no
Click chemistry [54,55]	no	yes	yes	yes	yes	yes
DNA-directed [56,57]	yes ${}^{\circ }$	yes	yes	yes	no	yes
Biotin-SAV [38]	no ${}^{\circ }$	yes	no ${}^{\circ }$	yes	no	yes
Protein A/G [58]	no ${}^{\circ }$	no	no ${}^{\circ }$	yes	no	no ${}^{\circ \circ }$
Carboxymethyl dextran [42]	no	no	yes	yes	yes	no
Thiol-gold coupling [59]	no	no	no	yes	yes	yes
Nanostructures [43]	no	no	no	yes	yes	no

${}^{\circ }$ Immobilization of streptavidin/protein A/protein G might need amide-coupling and/or be immobilized with a chemistry that forms a tridimensional structure. ${}^{\circ \circ }$ Probe might need amine-modification in case of absence of amine groups (etc. DNA).

**Table 2:** Features of the surface chemistry methods discussed here, ranked from * to ****, where a higher number of asterisks indicates a better performance.

Immobilization Strategy	Molecular Structure Preservation	Loading Capacity	Surface Morphology	Non-Specific Interactions	Accessibility
Physical adsorption [38]	**	*	**	*	****
Epoxysilane [38,39]	*	**	***	***	***
Copoly(DMA-NAS-MAPS) [40]	****	***	****	****	**
Dendrimers [50]	***	***	***	****	*
Hydrogels [51,52,53]	****	****	**	***	*
Click chemistry [54,55]	****	***	****	****	**
DNA-directed [56,57]	****	****	**	****	*
Biotin-SAV [38]	***	**	**	****	***
Protein A/G [58]	**	**	***	**	**
Carboxymethyl dextran [42]	****	****	**	***	**
Thiol-gold coupling [59]	*	**	****	**	***
Nanostructures [43]	**	***	**	****	*

## Data Availability

The study did not report any data.
